# GGDonto ontology as a knowledge-base for genetic diseases and disorders of glycan metabolism and their causative genes

**DOI:** 10.1186/s13326-018-0182-0

**Published:** 2018-04-18

**Authors:** Elena Solovieva, Toshihide Shikanai, Noriaki Fujita, Hisashi Narimatsu

**Affiliations:** 1 Glycoscience and Glycotechnology Research Group, National Institute of Advanced Industrial Science and Technology (AIST), Tsukuba, Japan; 2 GlycoBiomarker Leading Innovation Co. Ltd. (GL-i), Tsukuba, Japan

**Keywords:** Semantic web technologies, Ontology, RDF/SPARQL-based user interface, Glycan metabolism, Genetic diseases and disorders

## Abstract

**Background:**

Inherited mutations in glyco-related genes can affect the biosynthesis and degradation of glycans and result in severe genetic diseases and disorders. The Glyco-Disease Genes Database (GDGDB), which provides information about these diseases and disorders as well as their causative genes, has been developed by the Research Center for Medical Glycoscience (RCMG) and released in April 2010. GDGDB currently provides information on about 80 genetic diseases and disorders caused by single-gene mutations in glyco-related genes. Many biomedical resources provide information about genetic disorders and genes involved in their pathogenesis, but resources focused on genetic disorders known to be related to glycan metabolism are lacking. With the aim of providing more comprehensive knowledge on genetic diseases and disorders of glycan biosynthesis and degradation, we enriched the content of the GDGDB database and improved the methods for data representation.

**Results:**

We developed the Genetic Glyco-Diseases Ontology (GGDonto) and a RDF/SPARQL-based user interface using Semantic Web technologies. In particular, we represented the GGDonto content using Semantic Web languages, such as RDF, RDFS, SKOS, and OWL, and created an interactive user interface based on SPARQL queries. This user interface provides features to browse the hierarchy of the ontology, view detailed information on diseases and related genes, and find relevant background information. Moreover, it provides the ability to filter and search information by faceted and keyword searches.

**Conclusions:**

Focused on the molecular etiology, pathogenesis, and clinical manifestations of genetic diseases and disorders of glycan metabolism and developed as a knowledge-base for this scientific field, GGDonto provides comprehensive information on various topics, including links to aid the integration with other scientific resources. The availability and accessibility of this knowledge will help users better understand how genetic defects impact the metabolism of glycans as well as how this impaired metabolism affects various biological functions and human health. In this way, GGDonto will be useful in fields related to glycoscience, including cell biology, biotechnology, and biomedical, and pharmaceutical research.

**Electronic supplementary material:**

The online version of this article (10.1186/s13326-018-0182-0) contains supplementary material, which is available to authorized users.

## Background

Glycoscience refers to the study of the structures and functions of glycans and glycoconjugates and covers a wide range of topics, including the role of carbohydrates in disease development. Glycans play essential roles in many cellular functions and biological processes, and abnormalities in their metabolism lead to impaired functions of multiple organ systems and eventually result in the development of diseases and disorders. Inherited mutations in glyco-related genes can affect the biosynthesis of glycans as well as their degradation, and this impaired metabolism can result in severe genetic diseases and disorders, such as congenital disorders of glycosylation (CDG) and lysosomal storage diseases (LSD).

Genetic diseases and disorders of glycan metabolism are currently the subject of research in many scientific fields, including glycoscience and biomedical research. Many relevant information resources and databases are now publicly available, including the Online Mendelian Inheritance in Man (OMIM) [1], the Genetics Home Reference [2], the Genetic and Rare Diseases Information Center (GARD) resources [3], the Genetic Testing Registry (GTR) [4], and the Orphanet portal [5], among others. These resources provide information on a wide range of known hereditary diseases and disorders as well as their causative genes, and, moreover, their contents are not restricted to certain types of metabolic products. In contrast to these broadly focused information resources, the Glyco-Disease Genes Database (GDGDB) [6] has been created by the Research Center for Medical Glycoscience (RCMG) to provide information about hereditary diseases and disorders caused by defects of single glyco-related genes. The GDGDB provides detailed information about inborn errors of glycan metabolism in the context of their responsible genes and pathogenetic processes. It is a relational database with a web-based user interface and is available in English and Japanese.

Semantic Web technologies allow us to describe and organize scientific information, to create a knowledge-base for a particular scientific field, to share information across services, and to build links between related information resources. They have been widely applied to biomedical and life sciences information in recent years.

As a part of the “Life-Science Database Integration Project” of the National Bioscience Database Center (NBDC) sponsored by the Japan Science and Technology Agency (JST), we developed a knowledge-base of genetic diseases and disorders of glycan metabolism and their causative genes. We created an ontology named the Genetic Glyco-Diseases Ontology (GGDonto) [7]. For its development, we performed the following steps: 1) we created the schema and main content of GGDonto; 2) organized the GDGDB information, RDFized it, and integrated it into GGDonto; 3) added information about other genetic diseases and disorders sharing a similar etiology; 4) created a classification scheme for the diseases recorded in GGDonto; and 5) enriched the content of this ontology by linking it to other biomedical resources and integrating the information into GGDonto.

Our GGDonto ontology is based on Semantic Web technologies and is thus represented in Resource Description Framework (RDF) format using the RDF Schema (RDFS), Simple Knowledge Organization System (SKOS), and Web Ontology Language (OWL) vocabularies. We also developed a RDF/SPARQL-based user interface, which allows users to navigate the ontology, perform searches, and view detailed information in a user-friendly manner. These ontology and RDF/SPARQL-based user interface are available at http://acgg.asia/db/diseases/.

In this article, we introduce the topics of GGDonto, describe the structure of this ontology, and explain the functionality of its user interface, including browsing, searching, and filtering functionalities.

## Current knowledge on genetic diseases and disorders of glycan metabolism and their causative genes

Over the past several decades, associations between many human genetic disorders and mutations in genes involved in the biosynthesis and degradation of glycans have been newly identified [8–11].

### Current knowledge on genetic diseases and disorders of glycan biosynthesis

Glycosylation is the enzymatic process by which glycans are created, altered, and attached to proteins and lipids [11]. Genetic defects in glycosylation lead to a variety of inherited metabolic disorders known as CDG [10, 12–14]. In the last decade, several disorders of *N*-linked protein glycosylation, *O*-linked protein glycosylation, and glycolipid and glycosaminoglycan biosynthesis have been newly described, and this process of discovery is just beginning [11, 13–17]. While many glycosylation disorders are caused by defects in the *N*-glycosylation pathway, others result from defects in the *O*-glycosylation pathway, both the *N*- and the *O*-glycosylation pathways, or other pathways. However, defects in *C*-glycosylation have not yet been reported [13, 15]. Obviously, the discovery and detailed descriptions of novel CDG provide new knowledge about the role of glycans in human physiology and health and demonstrate a wide range of biological processes that are dependent on proper glycosylation [16].

The traditional classification of CDG was proposed at the First International Workshop on CDG and Related Disorders in Leuven in 1999; it is based on the serum transferrin pattern obtained by the isoelectric focusing test [11, 15, 18, 19]. In this classification, CDG are divided into two groups, I and II, and lowercase letters are used to indicate subtypes of disorders in chronological order of their discovery. In this classification, the CDG-I group includes disorders characterized by the under-occupancy of *N*-glycosylation sites, and the CDG-II group includes disorders caused by defects of *N*-glycan trimming and elongation [10, 13, 19, 20]. Based on the transferrin isoelectric focusing analyses, the traditional nomenclature has included only *N*-glycosylation disorders. *O*-Glycosylation and glycolipid biosynthesis disorders have not been included in this classification, and they have been assigned trivial or biochemical names not associated with the family of CDG [18, 19].

As the number of CDG disorders increased, the traditional classification became more complex and difficult. Moreover, this classification does not indicate the defective genes and enzymes that are responsible for the development of disorders [14, 15, 18]. In 2008, Jaeken et al. proposed a new nomenclature for all types of CDG [18], and in 2009, the traditional classification of CDG was revised [15]. In the new nomenclature, the name “Congenital Disorders of Glycosylation (CDG)” is used not only for all types of protein glycosylation disorders, but also for lipid glycosylation disorders [15, 18, 21]. In this nomenclature, glycosylation disorders are named by the official symbol of defective genes, followed by the abbreviation CDG. If the disorder had a letter-based name in the previous classification, this name is also used and follows in parentheses. For example, in the new nomenclature, CDG type Ia is named “PMM2-CDG (CDG-Ia)” [12, 13, 15, 18].

Disorders that are caused by defects of glycosylation have broad, diverse, and severe clinical phenotypes and underline the significant roles of glycosylation in human cells and tissues [11–14]. These clinical features are highly variable, ranging from infantile lethality to moderate intellectual disabilities in adults [14, 17, 19, 20]. Impaired glycosylation usually has a pathophysiological impact in multiple organ systems, but is particularly likely to affect brain development and functions of the nervous, musculoskeletal, digestive, and immune systems [10, 19]. Clinically, most patients with these disorders have a general failure to thrive, developmental delay, psychomotor retardation, neurological and neuromuscular impairments, and variable features, like musculoskeletal and eye abnormalities, coagulopathies, hormone dysfunction, and many other symptoms [10, 17, 19, 20]. However, the phenotypes of some glycosylation disorders are not completely understood and their clinical descriptions are limited owing to the small number of reported cases [12, 19].

Patients with each of these disorders are characterized by diverse clinical phenotypes, and therefore the clinical features cannot be used to identify a mutated gene [13, 19]. However, similar clinical features can point to common causes and common underlying mechanisms, and phenotypic similarities may be helpful for the identification of additional genes related to glycan biosynthesis and modifications [16]. Moreover, the discovery of novel glycosylation disorders with unexpected clinical findings as well as the description of clinical variability with additional findings for known disorders may help to understand the functions of glyco-related genes [13, 16]. Certainly, an increased knowledge of glycosylation disorders will contribute to a better understanding of the mechanism and impact of genetic defects on glycosylation and also the impact of glycosylation on various biological functions and human health [13, 14, 16, 20].

### Current knowledge on genetic diseases and disorders of glycan degradation

Inherited genetic defects that result in the absence or deficiency of specific lysosomal hydrolases cause LSD. Missing or deficient enzymes cause the accumulation and storage of intermediate compounds in cells, tissues, and organs, and the macromolecules that are not properly degraded lead to cellular damage and the onset of symptoms [9, 22–24].

Most glycans are degraded and recycled in lysosomes [9, 23, 25]. In this manuscript, we discuss LSD that are associated with the impaired degradation of glycans and glycoconjugates. These LSD are generally classified according to the type of glycoconjugate whose catabolism is impaired, and they are usually divided into three groups [9, 23]. The first group contains diseases caused by genetic defects in enzymes that are involved in the lysosomal degradation of oligosaccharide chains of glycoproteins. These diseases are also called “oligosaccharidoses.” The second group contains diseases associated with genetic deficiencies in lysosomal enzymes that degrade the polysaccharide chain (glycosaminoglycan) of proteoglycans. These types of diseases are called “mucopolysaccharidoses” (MPS). The third group contains diseases associated with genetic defects in enzymes that are involved in glycolipid degradation. These types of diseases are called “sphingolipidoses” when they are associated with the catabolism of sphingolipids and “other lipid storage diseases” when they are generally not classified as sphingolipidoses, such as Wolman disease, which affects the metabolism of cholesteryl esters and triglycerides [9, 23, 26].

Glycan-related LSD are characterized by a progressive multisystem pathology and a variety of progressive physical impairments and mental deterioration [22, 24]. The phenotypes associated with these LSD include the following clinical characteristics: brain pathology with central nervous system manifestations, hepatomegaly, splenomegaly, skeletal abnormalities, and heart and lung pathology [2, 24]. For many LSD, different degrees of severity may be present. If genetic defects result in the complete obliteration of enzyme activity, affected individuals tend to have earlier onsets of symptoms. If genetic defects result in the significant reduction of enzyme activity, clinical signs and symptoms may manifest later in childhood, adolescence, or adult life, and patients with these juvenile, childhood, or adult onsets usually display more moderate symptoms than those of patients with earlier onsets [9, 22]. Moreover, the organ systems involved may differ with respect to the time of onset [9].

Inherited genetic defects in the lysosomal catabolism of glycans show the importance of the balance between glycan synthesis and degradation for proper biological functions of cells and tissues [25, 27]. The lysosomal degradation of glycans and glycoconjugates are ordered and highly specific processes and many new insights in our understanding of these complex pathways have been obtained in studies of LSD [9].

## Methods

As described above, both glycan biosynthesis and lysosomal catabolism are very complicated and highly regulated processes, playing important roles in tissue homeostasis. For a better understanding of the etiology and clinical characteristics of these diseases and disorders, more comprehensive information is needed, and this is the aim of GGDonto.

### Designing the structure and content of the GGDonto ontology

To provide comprehensive information about genetic diseases and disorders of glycan metabolism, we designed the structure and content of GGDonto.

Most of the illnesses may be described by various characteristics, including their names, etiology, pathogenesis, clinical manifestations, nosological classifications, the descriptions and codes from coding systems, and identifiers from biomedical sources. We used the same approach to describe the diseases and disorders included in GGDonto. Later in this section, we have described the types of information included in the content of GGDonto, the classes and properties used for data representation, and the sources that were used to obtain the information for ontology creation.

As the metabolism of glycans is very complicated, for the development of a knowledge-base of genetic diseases and disorders caused by defects in this metabolism, it was necessary to describe these impairments in detail in the context of metabolic pathways. Moreover, because these impairments lead to significant clinical manifestations, it was important to organize phenotype-related information in a way that is easy to navigate and understand. For this purpose, we decided to create classifications of the GGDonto diseases and disorders.

In nosology (the branch of medical science dealing with the classification of diseases), diseases are usually classified by etiology (cause), pathogenesis (mechanism by which the disease is caused), the presenting symptoms, or the organ systems involved. We also designed and created similar types of classifications of the genetic diseases and disorders of glycan metabolism.

The causes of all diseases and disorders included in GGDonto are single-gene mutations in glyco-related genes that result in the absence or deficiency of enzymes involved in the metabolism of glycans. As a consequence, the mechanism underlying these diseases and disorders is associated with impaired glycan metabolism, which leads to glycan deficiency or accumulation. As the cause and mechanism are both associated with the metabolic pathways of glycans, we decided to design the “Pathway” classifications that represent the etiological and pathogenetic aspects of the GGDonto diseases and disorders. These “Pathway” classifications are the main advantage of our ontology. We designed this type of classifications on the basis of scientific literature, and to our knowledge, this is the first time classifications with such a structure and content, including all subcategories, are being presented in an ontology. The details of the structure of our “Pathway” classifications are presented in Additional file 1.

Along with “Pathway” classifications, we designed “Phenotype” classifications that grouped diseases and disorders by their symptoms and involved organ systems. All of these classifications have been described in the subsection “Classifications of the GGDonto diseases and disorders.”

To provide more detailed knowledge about the cause and mechanism, symptoms, and phenotypes of the GGDonto diseases and disorders, we decided to add the corresponding information from related biomedical resources, such as various descriptions of causative genes and corresponding enzymes, and definitions of diseases and phenotypic characteristics. In the subsection “Linking GGDonto to related biomedical resources”, we have explained how these resources were integrated into the GGDonto ontology.

### Application of semantic web technologies for the development of the GGDonto ontology

The GGDonto ontology was developed for the particular subject domain using the Semantic Web standards, such as RDF, RDFS, and SKOS in combination with OWL. OWL was used to represent the semantic structure and semantic relations of this ontology. To describe the information in GGDonto, we used many elements from the OWL vocabulary as well as our self-defined classes and properties that were also defined by rdf:type property as instances of owl:Class or owl:ObjectProperty and owl:DatatypeProperty.

To organize distinct concepts (ideas or meanings) of the GGDonto ontology into concept schemes (a set of concepts that may include hierarchical, associative, and semantic relationships between them) we used the SKOS vocabulary. As it is described in the SKOS Reference (https://www.w3.org/TR/skos-reference/) and SKOS Primer (https://www.w3.org/TR/skos-primer/), the notion of a concept scheme is useful when dealing with two or more different knowledge organization systems, such as thesauri, taxonomies, classification scheme, and subject heading systems. We created the instance of skos:ConceptScheme class for each of our classifications, in accordance with the fact that the origins of their structure are different information sources, including the Medical Subject Headings (MeSH), UMLS Metathesaurus, and scientific literature that describes the classifications of CDG and LSD. Moreover, the information from other sources, such as the GDGDB database and some of the NCBI databases, is also included in the content of our system. For each of these, we also created the instance of the skos:ConceptScheme class.

We defined the main elements (meanings) of our ontology, such as the diseases, symptoms, or terms in the disease classifications as instances of skos:Concept class, and their semantics as instances of our self-defined classes. Using the skos:inScheme property we linked each of these concepts with one or more of our concept schemes. Because in our content each of the diseases or disorders is included in two (“Pathway” and “Phenotype” for LSD) or three (traditional and new “Pathway” and “Phenotype” for CDG) classifications, each disease concept is linked with two or three of our concept schemes. As it is described in the SKOS Primer, for the SKOS concepts, it is possible to participate in several concept schemes at the same time. Moreover, by using the skos:broader, skos:narrower, and skos:related properties, as well as their subproperties defined in GGDonto, we aggregated the set of concepts from each of our schemes into distinct structures with their own hierarchical, associated, and semantic relationships.

As we organized our information into multiple concept schemes, it was necessary to indicate how these concepts are related to each other. For this purpose, along with using semantic relation properties defined in our ontology, we used the SKOS mapping properties, such as skos:closeMatch and skos:exactMatch. As it is described in the SKOS Primer, the SKOS mapping properties helped us to indicate that two concepts from different schemes have comparable meanings, and to specify these meanings. Linking concepts by mappings (skos:closeMatch and skos:exactMatch) and the possibility of a concept to participate in different concept schemes (skos:inScheme) are the important features and key advantages of SKOS. This approach helps avoid the taxonomical conflicts that may be occurring through the integration different information sources. For example, CDG are classified in our ontology by the traditional and new “Pathway” classifications at the same time.

#### Creating the schema and main content of the GGDonto ontology

To provide comprehensive information about genetic diseases and disorders of glycan metabolism, defined these genetic diseases and disorders as the main elements (concepts) of our ontology. The GGDonto ontology was developed on the basis of the scientific literature and information resources, and the corresponding references were included in each concept of the ontology using the “dct:references” property. We used self-defined classes and properties as well as the classes and properties from existing publicly available RDF vocabularies.

To define classes, properties, and instances, we created the schema for GGDonto. We specified the namespace for this schema as <xmlns:ggdsch = “http://jcggdb.jp/rdf/diseases/ggdonto-schema%23”> with the prefix “ggdsch” [see Additional file 2]. Additionally, we created the schema with the prefix “gmsch” and the namespace <xmlns:gmsch = “http://jcggdb.jp/rdf/diseases/gmncbi-schema%23”>. In this schema, we defined classes and properties for describing information from NCBI databases. We used the information from the NCBI databases for the purpose of enriching the content of GGDonto.

We defined the main structure of our ontology using the “Concept” class from the SKOS vocabulary, and their instances are concepts that represent the diseases, clinical findings, or terms in the disease classification. The “diseases” concepts describe the diseases and disorders, and the “clinical findings” concepts describe the symptoms, signs, and abnormal clinical and laboratory findings. We created the relationships from the “diseases” concepts to “clinical findings” concepts using the gmsch:hasManifestation object property; in the opposite direction, we used the gmsch:manifestationOf object property. The “classifications” concepts were used to create the “Pathway” and “Phenotype” classifications. In order to describe the semantic relations, we defined the ggdsch:SemanticRelationsGGDonto class, identified as a subclass of the owl:ObjectProperty class, and many of its instances were used for the creation of these classifications.

Most of the concepts in GGDonto that represent diseases and disorders, signs and symptoms, and various processes in metabolic pathways are mapped to corresponding concepts in the Unified Medical Language System (UMLS) Metathesaurus [24]. In our ontology, we indicate these concepts in UMLS by their Concept Unique Identifiers (CUI). For some of the concepts in our ontology, we also included other identifiers from various sources of the UMLS, such as the “Unique Identifier” or “Code” for the corresponding concepts. Moreover, for the concepts that represent the diseases and disorders, the unique identifiers from the Online Mendelian Inheritance in Man (OMIM) [1], i.e., the phenotype MIM number and gene MIM number, were also included in GGDonto.

Causative genes of genetic diseases and disorders of glycan metabolism are also the main topics of GGDonto. However, we do not describe them within the GGDonto ontology. Instead, we used the entries from NCBI’s Gene [28] and ClinVar [29] databases. We used the corresponding data from [Homo_sapiens.gene_info] and [gene_condition_source_id, mim2gene_medgen] files and RDFized them. These RDF versions of data obtained from NCBI contain the gene descriptions as well as information on the relationships between MIM numbers (OMIM), GeneIDs, and records in the MedGen database [30]. We created the relationships between “diseases” concepts in the GGDonto ontology and “genes” concepts in the RDF representation of NCBI’s Gene [28] and ClinVar [29] data using the gmsch:causedBy object property. An additional RDF file shows all data included in the GGDonto ontology [see Additional file 3].

To use the classes and properties from publicly available vocabularies, we included the base URLs of their namespaces in the schema of our ontology. The namespaces of specifications, such as RDF, RDFS, SKOS, and OWL, indicate Semantic Web standards that are often used for ontology creation. The namespaces of vocabularies, such as DCT (Dublin Core Metadata Terms), BIBO (Bibliographic Ontology), PRISM (Publishing Requirements for Industry Standard Metadata), PAV (Provenance, Authoring and Versioning), and FOAF (Friend of a Friend), indicate the vocabularies and standards used to describe the elements, such as metadata, references, related resources, and the identifiers of the RDF resources in our ontology.

#### Creating “GDGDB RDF” by RDFizing the GDGDB data

The content of GDGDB can be divided into two groups: 1) information about diseases and disorders and 2) information about their causative genes. The information about each of the diseases and disorders includes the following elements: the name and synonyms of the disease, the pathogenesis of the disease, the phenotype from OMIM, and the phenotype MIM number from OMIM. The information about each of the causative genes includes the following elements: the official and synonymous symbols of the gene, the name and synonymous names of the gene, the chromosome location, and the gene ID from the NCBI Gene database.

In accordance with the semantics of GDGDB, we designed the structure of its RDF version and named this version “GDGDB RDF.” This version of GDGDB is represented in RDF/XML syntax using the RDFS, SKOS, and OWL vocabularies. We defined the namespace for the schema of “GDGDB RDF” with the prefix gdgsch: <xmlns:gdgsch=http://jcggdb.jp/rdf/diseases/gdgdb-schema%23> [see Additional file 4].

To describe the information about the diseases and disorders and about the glyco-related genes, we created two main classes: “DiseaseDescription” and “GeneDescription.” We described the diseases and disorders using the skos:Concept class, and one instance of this class was created for each. The “Document ID” from the GDGDB was used as an identifier for each “diseases” concept as well as to link to the GGDonto ontology. To describe the semantics of each concept, we defined the following properties: diseaseName, pathogenesis, omimPhenotype, omimPhenoMIMnumber, geneSymbol, aliasGeneSymbol, geneName, aliasGeneName, locus, and ncbiGeneId. The meaning of these properties corresponds to the elements of the information of GDGDB. For example, the locus property is used to describe the chromosome location of the causative genes included in GDGDB. Using these classes and properties, we RDFized the GDGDB data. An additional RDF file shows all data included in “GDGDB RDF” [see Additional file 5].

Since the development of a knowledge-base of genetic diseases and disorders of glycan metabolism included almost all of the diseases and disorders known to be associated with this pathogenesis, we decided to develop the ontology with a wide range of information related to these aspects of glycoscience.

#### Additional diseases and disorders with similar etiologies

At present, GGDonto provides information on 120 diseases and disorders of glycan metabolism, which is 40 more than the number included in the original GDGDB database. All of the additional disorders are related to the synthesis of glycans, and they can all be classified as CDG. These disorders are not included in the GDGDB database because they are extremely rare or they have been identified as disorders related to glycan metabolism in just the last few years. For example, disorders caused by defects in the COG complex, such as COG1-CDG, COG4-CDG, COG5-CDG, OG6-CDG, and COG8-CDG, have been identified in recent years [31–33]. New diseases are constantly being discovered, and those that are related to genetic defects in glyco-related genes can be easily added to GGDonto by adding the corresponding concepts to our ontology.

#### Classifications of the GGDonto diseases and disorders

To describe the semantics of the information related to diseases and disorders, we divided them into two groups according to the type of abnormal metabolic processes: synthesis or degradation. For each of these two groups, we designed and constructed two types of classifications, referred to as “Pathway” and “Phenotype” classifications.

The “Pathway” classifications are used to represent and describe the etiology and pathogenetic aspects of the diseases and disorders by describing the impaired metabolic processes. “Pathway” classifications were constructed on the basis of the scientific literature and widely used classifications [11, 15, 18, 19, 21], as mentioned in a previous section, “Current knowledge on genetic diseases and disorders that affect glycan metabolism.”

The “Phenotype” classifications are used to represent and describe the clinical features and clinical phenotypes of the diseases and disorders by describing the symptoms, signs, and abnormal clinical and laboratory findings. Most of the medical terms used in our “Phenotype” classifications are defined and described in the UMLS Metathesaurus [24]. All of these terms have unique identifiers (CUI), which we also included in our ontology with the corresponding terms.

All of the classifications have a hierarchical structure. The structures of the “Pathway” classifications correspond to the structures of published and widely used classifications [9, 11, 15, 18, 23, 34]. In addition, we checked these classifications by comparisons with information from other publications [14, 19, 21, 25]. The structures of the “Phenotype” classifications are based on the “Vocabulary Terms” and “Tree Structures” of the Medical Subject Headings (MeSH) [35]. Moreover, we integrated all of these “Pathway” and “Phenotype” classifications into a single classification tree and applied this tree to all genetic diseases and disorders included in GGDonto. All of the classifications were assigned manually.

To browse the hierarchical structure of these classifications and view the data associated with each term, we have developed a web-based tool [36]. This tool provides all information from the GGDonto ontology, excluding information from “GDGDB RDF” and other resources. This tool has browsing and link features, but has no ability to search data and to link it with other resources through Semantic Web technologies. For this purpose, we developed a RDF-based user interface, and its functionalities are described in detail later.

#### Linking GGDonto to related biomedical resources

Semantic Web technologies enable us to link our information with related information from other scientific resources. We used these technologies to create a comprehensive knowledge-base for the field of glycoscience. In particular, we used a Semantic Web approach to include a range of related information in the knowledge-base and to enable access to this information via a user-friendly interface.

To describe the diseases, disorders, and their causative genes in a comprehensive manner, we used information from the NCBI MedGen [30], Gene [28], and ClinVar [29] databases. Because this information was not yet represented in RDF format, we RDFized it, defined the namespaces for these data, and uploaded the RDF files into our SPARQL Endpoint. Finally, this information is viewed and retrieved through the RDF/SPARQL-based user interface of GGDonto.

To include information on the proteins and enzymes that correspond to the genes associated with glycan metabolism, we used the information from “UniProt in RDF” [37]. Because this information is already represented in RDF format, we used it in its original RDF format. In our ontology, the information about the proteins, such as the UniProt Accession number, UniProt Entry name, Protein Recommended Name, Protein Function, and Catalytic Activity, was obtained from entries from the <https://sparql.uniprot.org/sparql/> data set in the UniProt Knowledgebase (UniProtKB). Information about the enzymes, such as the EC Number, Accepted Name, and Reaction catalyzed, was obtained from the <https://sparql.uniprot.org/sparql/> data set in UniProtKB. This enzyme-related data set corresponds the data available in the Swiss-Prot ENZYME database.

Moreover, we created links to the entries of GlycoGene DataBase (GGDB) (http://acgg.asia/ggdb2/), which was also developed by the Research Center for Medical Glycoscience (RCMG) and provides comprehensive information about glyco-related genes. The latest version of GGDB is also represented in RDF format; accordingly, we can access it using Semantic Web technologies.

### Development of the RDF/SPARQL-based user interface

Along with “GDGDB RDF” and the GGDonto ontology, we developed the RDF/SPARQL-based user interface [38], by which users can retrieve and extract information from content represented in RDF format. The basic structure of this user interface was developed in Java and JSP. For better programming efficiency, the Handlebars^1^ template was used to generate the web pages. To process Handlebars files, HandleBars.java, which is a Java implementation of Handlebars, was used. Moreover, to provide functionalities, such as the filtering, sorting, and browsing of faceted classifications, we used the Exhibit framework^2^, which is a JavaScript framework for large-scale data-rich interactive Web pages. For querying our RDF data, we used SPARQL statements. SPARQL (Simple Protocol and RDF Query Language) is a query language for RDF, and it is the W3C Recommendation [39]. These applications and implementations enable our data from multiple datasets represented in RDF to be browsed, filtered, sorted, and searched by users, giving them the ability to quickly find the target information.

## Results

### Full content of the developed knowledge-base

**Fig. 1:**
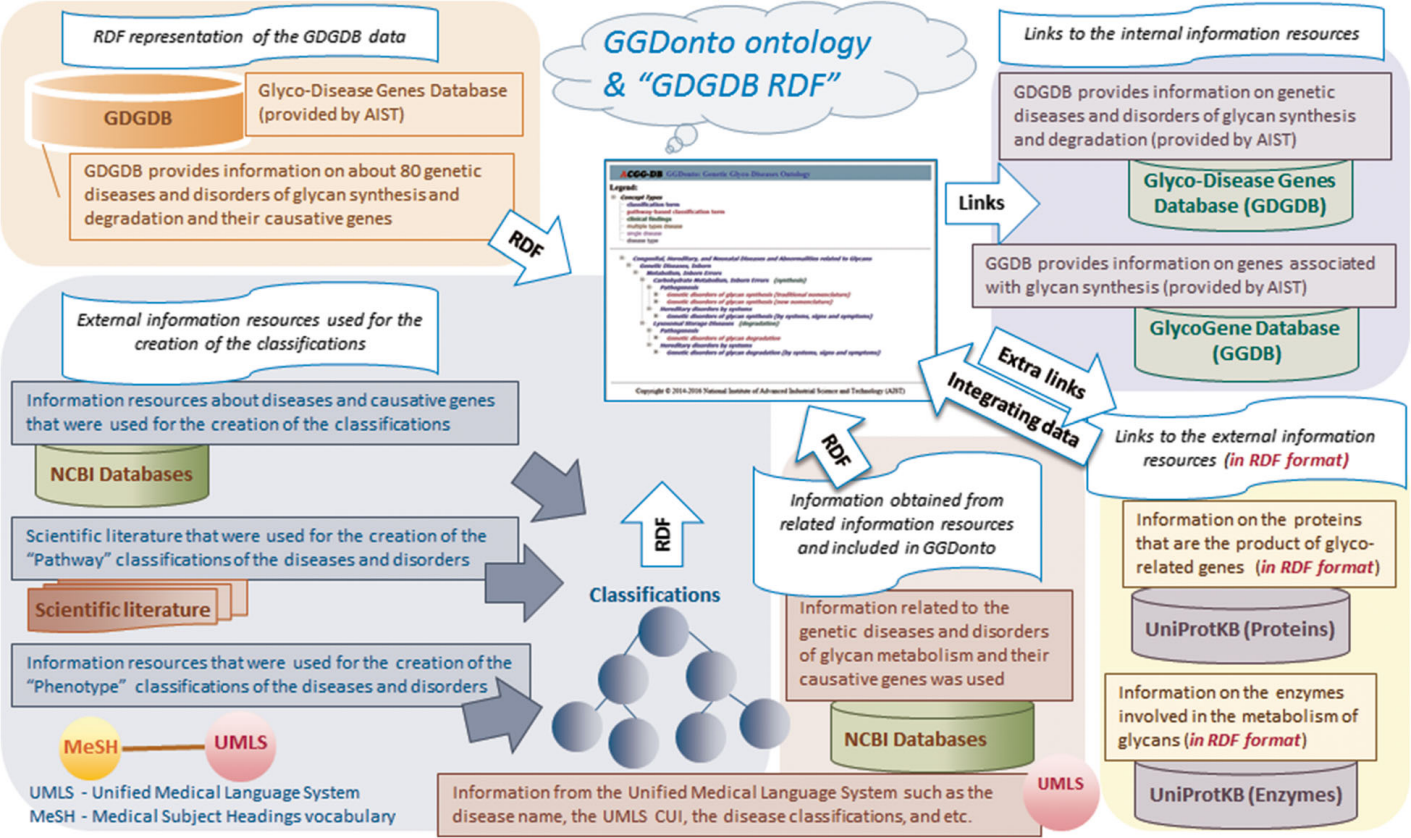
Internal and external information resources used for the creation of GGDonto and the related knowledge-base

The main content of the developed knowledge-base consists of the GGDonto ontology within which all entries are connected to the “diseases” concepts of GGDonto. Figure 1 shows the internal and external information resources used to create the GGDonto ontology and other components of the developed knowledge-base. In addition to the GGDonto ontology, which includes information about genetic diseases and disorders of glycan metabolism as well as their “Pathway” and “Phenotype” classifications, the full content of the developed knowledge-base is stored in “GDGDB RDF,” the entries from the NCBI databases [28–30], and the “UniProt in RDF” repository [37], and links to internal resources, such as the GDGDB and GGDB databases. A key difference between the entries from the NCBI databases and the “UniProt in RDF” repository is that the information from NCBI databases was not represented in RDF and therefore we RDFized it and integrated it into the developed knowledge-base. In contrast, information from the “UniProt in RDF” repository was already represented in RDF and accordingly was directly integrated into the developed knowledge-base.

**Fig. 2:**
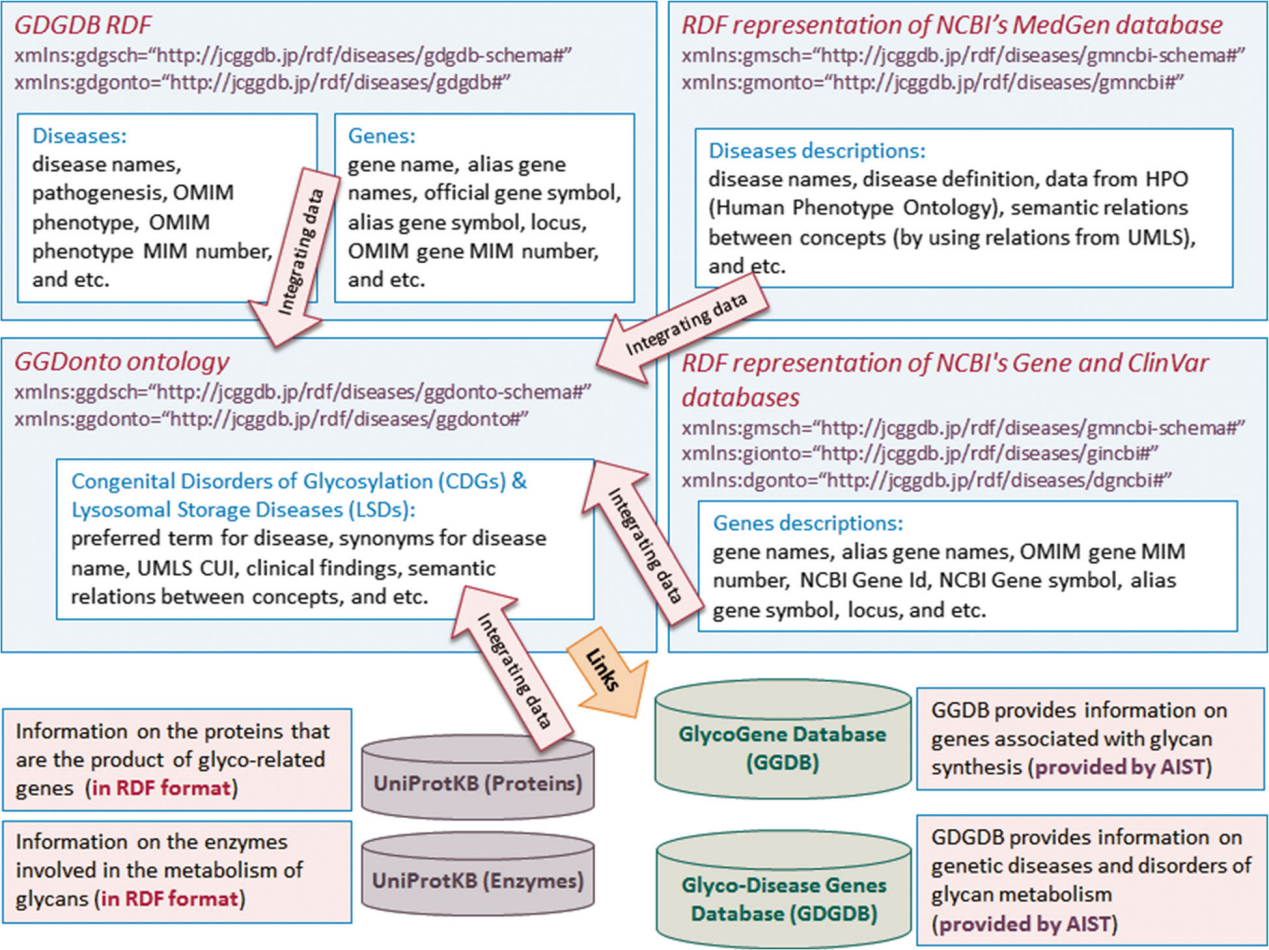
Summary of the content of the GGDonto ontology with the integrated datasets

In Fig. 2, we show the semantics of the content of the GGDonto ontology with the integrated datasets. The information provided includes the descriptions of genetic diseases and disorders of glycan metabolism and their causative genes as well as information on the proteins and enzymes associated with these causative genes. In addition to various descriptions, the content provided also includes various semantic relationships between multiple types of information. To summarize, the integration of information from multiple sources enriches the content of this knowledge-base, and this approach can be helpful for the effective retrieval of information stored in different sources and databases.

### Functionalities of the GGDonto user interface

To provide the information of the GGDonto ontology and integrated resources in the most effective way, we developed a RDF/SPARQL-based user interface. This user interface was developed using the Semantic Web technologies, including the SPARQL Endpoint and SPARQL queries. We registered all RDF data included in the created knowledge-base in a SPARQL Endpoint, and thus we could apply SPARQL queries to all of our information in order to browse and search it.

**Fig. 3:**
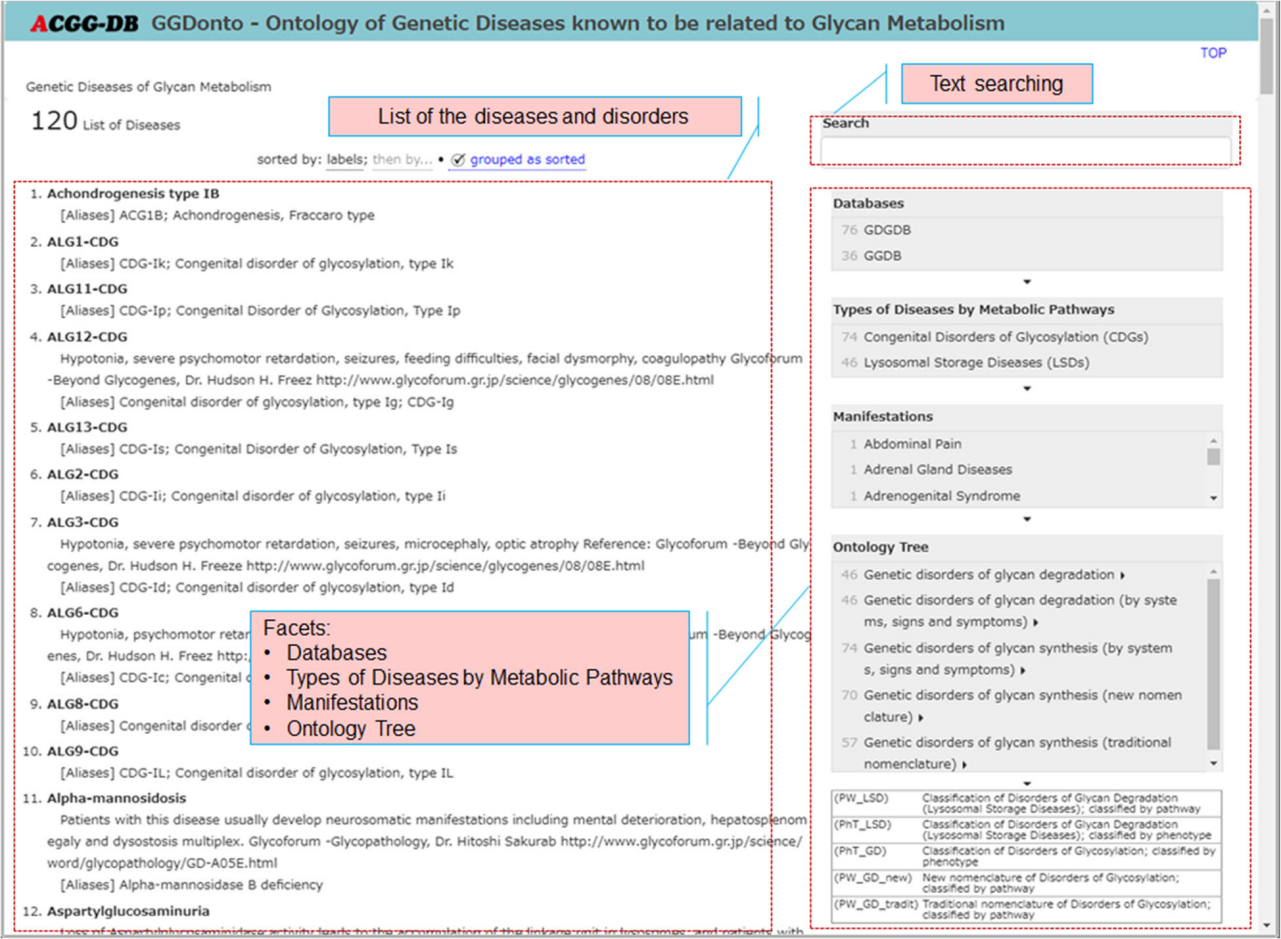
Top page of the RDF-based user interface

**Fig. 4:**
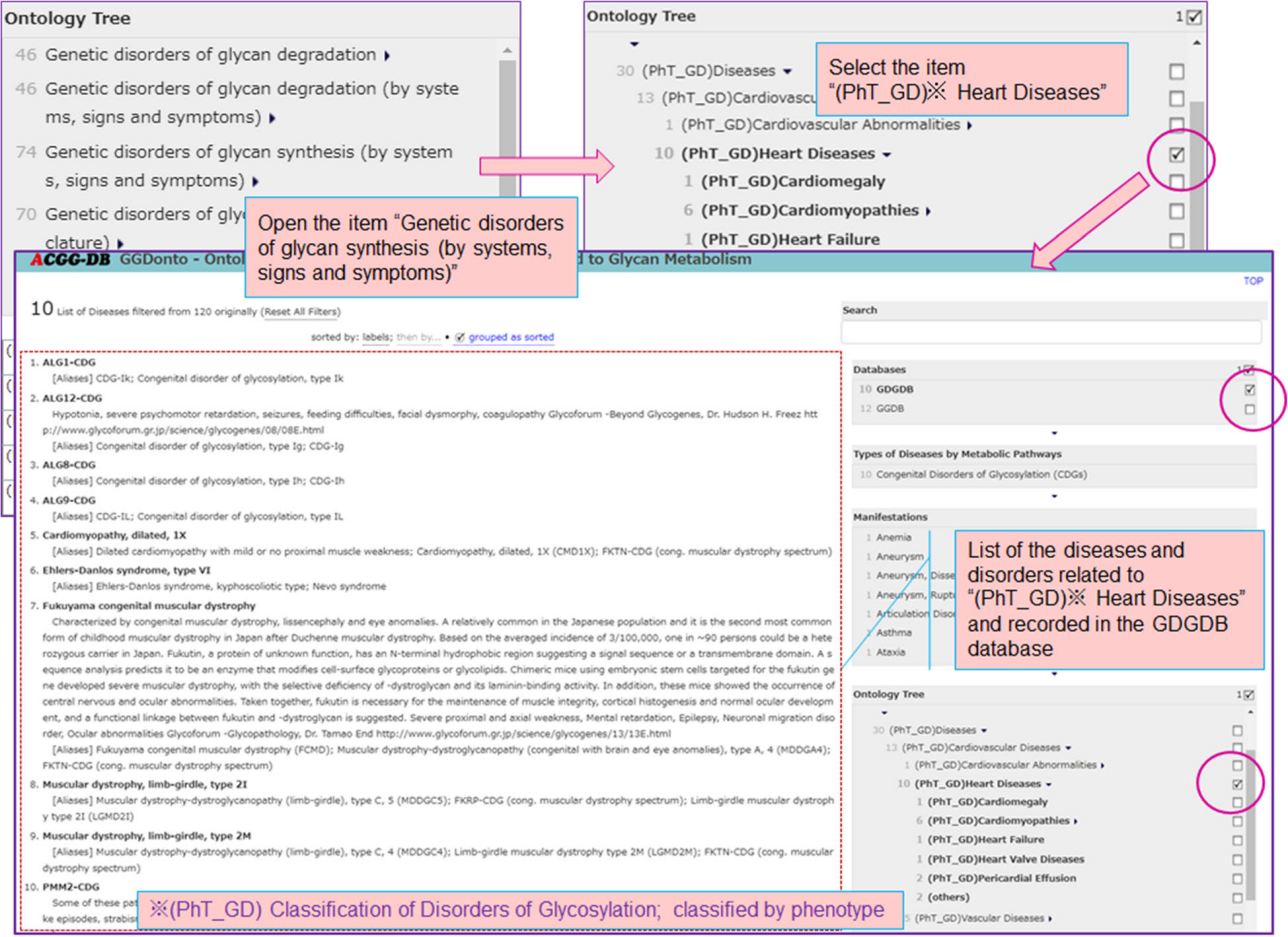
Example of the application of the Ontology Tree to narrow down the subset of the diseases and disorders

**Fig. 5:**
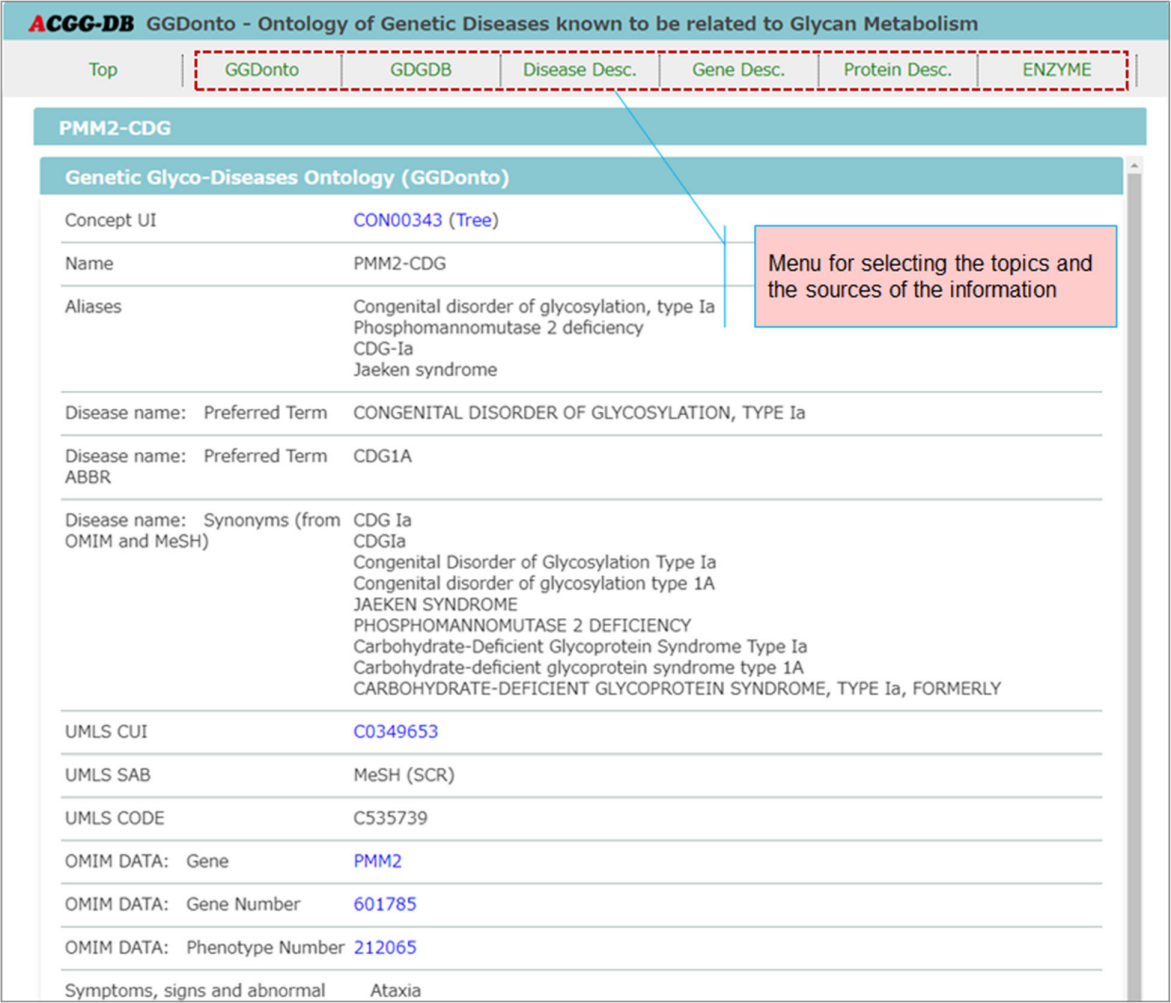
Page displaying detailed information about “Congenital disorder of glycosylation, type Ia” in the RDF-based user interface

**Fig. 6:**
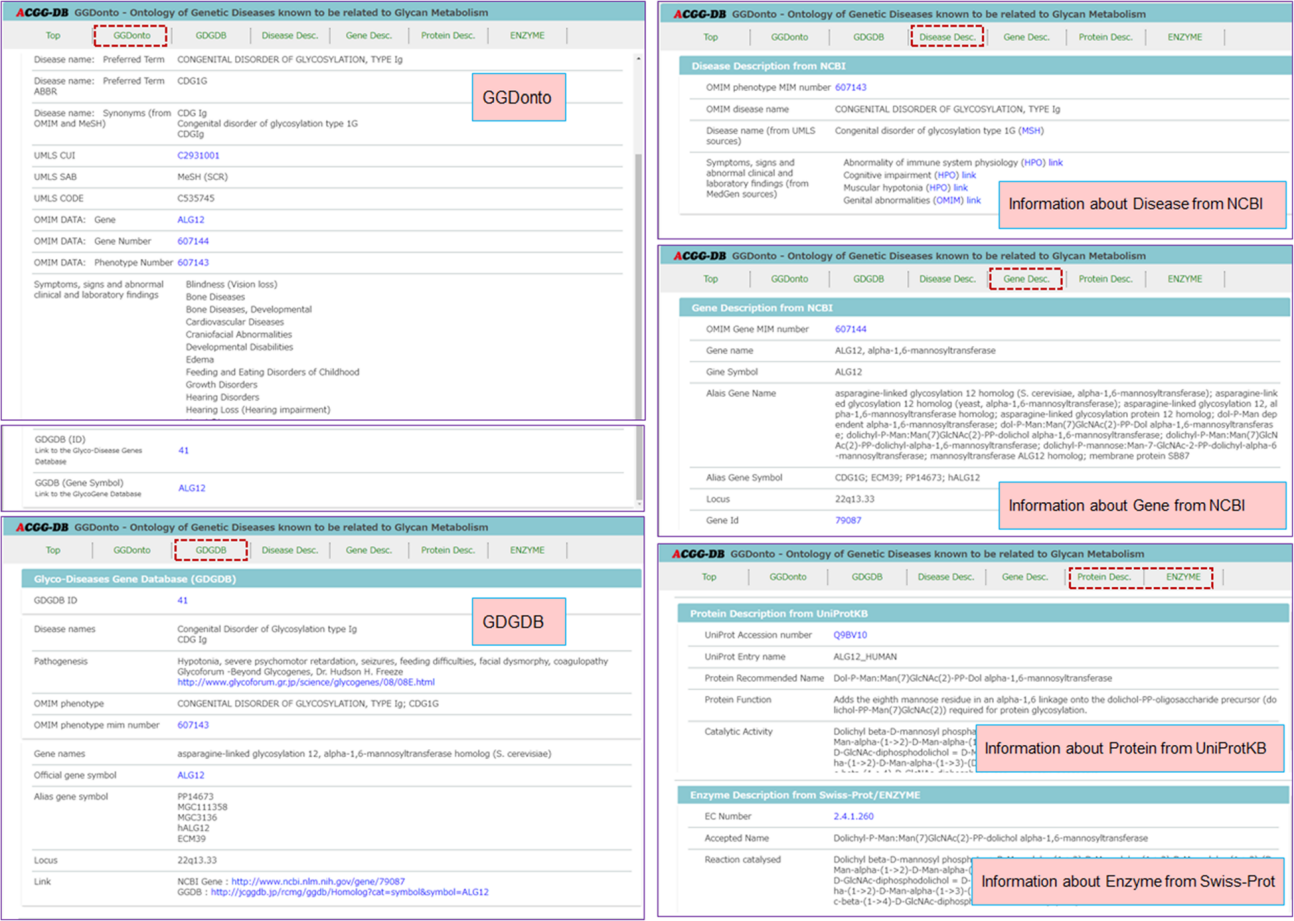
Topics and sources of information (from the page displaying detailed information about “Congenital disorder of glycosylation, type Ig”)

Figures 3, 4, 5, and 6 provide screenshots of the created user interface. In Fig. 3, we show the top page of this user interface. The GGDonto ontology is the main component of the developed knowledge-base; all of the information that is displayed here is from this dataset only. The list of genetic diseases and disorders of glycan metabolism that are recorded in the GGDonto ontology is shown on the left side of this figure. The text field for entering search strings is displayed on the top right corner. Text searching allows users to search across the information of the GGDonto ontology, including various descriptions and classifications of the diseases and disorders. As a result, only those diseases and disorders that are related to the search string are displayed.

The various facets for the faceted search (faceted navigation) are shown on the right side of Fig. 3. Each of these facets is based on the information that is included in the GGDonto ontology. These facets are as follows: 1) Databases (GDGDB and GGDB), for selecting the diseases and disorders that are recorded in the GDGDB or GGDB databases; 2) Types of Diseases by Metabolic Pathways (CDG and LSD), for selecting the diseases and disorders classified as CDG or LSD; 3) Manifestations, to narrow down the diseases and disorders that represent selected manifestations; 4) Ontology Tree, to narrow down the diseases and disorders that are related to the selected items in their classifications.

By choosing the facet filter items from multiple facets, users can display only the diseases that are associated with these selected items. This is an “OR” relationship between multiple facet filters. Because we used the selected items in manifestations and classifications of the diseases and disorders for faceted navigation, this allows users to better understand the semantics and the structure of the GGDonto ontology, and allows them to quickly find the information they are looking for.

In Fig. 4, we show an example of the application of the Ontology Tree to narrow down the subset of diseases and disorders being shown. In this example, the diseases and disorders are narrowed down by selecting the “(PhT_GD) Heart Diseases” item in the “Phenotype” classifications of the Congenital Disorders of Glycosylation. As result, a list of 10 disorders is displayed.

As an example, in Fig. 5, we show the page of detailed information about “Congenital disorder of glycosylation, type Ia” obtained by selecting this disorder in the list of genetic diseases and disorders on the top page. The menu for selecting the topics and sources of information is shown on the top side of Fig. 5.

Figure 6 shows a detailed view of Fig. 5. As shown in this figure, the information from NCBI MedGen, Gene, and ClinVar databases is used to describe the genetic diseases and disorders of glycan metabolism and their causative genes, while the information from the UniProtKB/Swiss-Prot repository is used to describe the related proteins and enzymes.

In short, the GGDonto user interface has various functionalities, including the browsing of faceted classifications, faceted navigation (faceted searching), and keyword searching. Certainly, by using this user interface, the complicated structure of the GGDonto ontology and integrated datasets can be quickly viewed, browsed, and searched by users, without requiring knowledge of RDF and SPARQL.

## Discussion

### Advantages of the GGDonto ontology

Using the advantages of Semantic Web technologies, we developed the GGDonto ontology as a knowledge-base for genetic diseases and disorders known to be related to glycan metabolism and their causative genes. Semantic Web technologies, such as RDF, RDFS, SKOS, OWL, and SPARQL, give us the opportunity to develop the ontology and link it to related information represented in RDF syntax. We implemented these Semantic Web technologies to represent our information in the RDF format and enrich the information included in the GDGDB database. As a result, GGDonto provides information on genetic diseases and disorders not only in the context of their pathogenesis and causative genes, but also in the context of related proteins, enzymes, and underlying metabolic processes. In this way, the GGDonto ontology may be used as a knowledge-base of the field in glycoscience.

To effectively provide the content of GGDonto, we developed the RDF/SPARQL-based user interface with various features, including a keyword search and faceted navigation. This user interface allows users to navigate the ontology tree, retrieve and search data, and find related information. By integration with other biomedical resources, related scientific information can be searched and viewed through our user interface, thus enabling users to easily find comprehensive information on their topic of interest.

Developed by the Glycoscience and Glycotechnology Research Group, GGDonto is linked to the GlycoGene DataBase (GGDB), which was developed by the same research group. The GGDB database is also represented in RDF format and provides comprehensive information about glyco-related genes. The link with the GGDB database provides users of GGDonto with a deeper knowledge of the causative genes of genetic diseases and disorders of glycan metabolism.

### Comparison with other information resources related to genetic diseases and disorders

Unlike many other information resources that provide information on a broad range of various genetic diseases and disorders, GGDonto is centered on those resulting from single-gene defects associated with the metabolism of glycans. This allows us to provide users with a comprehensive knowledge of this particular topic, including various classifications of the diseases and disorders.

Additionally, in contrast to many other information resources represented in common formats, such as relational databases, XML, CSV, or unstructured text, the GGDonto ontology is represented in RDF syntax, and its user interface is based on SPARQL queries. Accordingly, this Semantic Web approach has various advantages.

## Conclusions

Created by the RCMG and released in April 2010, GDGDB provides information on about 80 genetic diseases and disorders of glycan synthesis and degradation. For the purpose of providing more comprehensive information about these diseases and disorders in a more efficient way, we developed the GGDonto ontology and represented it using Semantic Web standards. At present, GGDonto provides information on 120 diseases and disorders with a wide range of related information. By providing integrated information on the pathogenesis and manifestations of these diseases and disorders with information on responsible genes, proteins, and enzyme activity associated with these genetic defects, GGDonto has the advantage of representing complex results in a more comprehensible manner.

GGDonto and “GDGDB RDF” have been published as a part of the information resources of the Asian Community of Glycoscience and Glycotechnology (ACGG) and are available on its website [7]. In addition, we have stored the GGDonto ontology in the NBDC RDF Portal [40], which provides a collection of life science datasets in RDF. The NBDC RDF Portal is managed by National Bioscience Database Center (NBDC) sponsored by the Japan Science and Technology Agency (JST). At present, the GGDonto ontology provides information on 120 genetic diseases and disorders with information on about 120 of their causative genes, and as can be seen on the “Statistics” page of the NBDC RDF Portal (https://integbio.jp/rdf/?view=matrix), GGDonto contains 1024 links, 23 classes, 1705 instances, 4963 literals, 1782 subjects, 943 properties, and 8978 objects.

We hope that GGDonto will be helpful for all researchers interested in all glycoscience, especially those interested in the pathogenesis and clinical features of genetic diseases and disorders known to be related to glycan metabolism. Moreover, we hope that the RDF/SPARQL-based user interface will be useful for obtaining a wide range of information related to this topic.

## Additional files

Additional file 1: Details of the structure of the “Pathway” classifications. (DOCX 161 kb)

Additional file 2: Schema of the GGDonto ontology (available at: http://jcggdb.jp/rdf/diseases/ggdonto-schema). (RDF 594 kb)

Additional file 3: Data of the GGDonto ontology (available at: http://jcggdb.jp/rdf/diseases/ggdonto). (RDF 2288 kb)

Additional file 4: Schema of “GDGDB RDF” (available at: http://jcggdb.jp/rdf/diseases/gdgdb-schema). (RDF 12 kb)

Additional file 5: Data of “GDGDB RDF” (available at: http://jcggdb.jp/rdf/diseases/gdgdb). (RDF 316 kb)

## Abbreviations

ACGG: Asian Community of Glycoscience and Glycotechnology
GDGDB: Glyco-Disease Genes Database
GGDB: GlycoGene DataBase
GGDonto: Genetic Glyco-Diseases Ontology
JST: Japan Science and Technology Agency
NBDC: National Bioscience Database Center
RCMG: Research Center for Medical Glycoscience
SKOS: Simple knowledge organization system
SPARQL: The simple protocol and RDF query language
UMLS: Unified Medical Language System

## Footnotes

1
http://handlebarsjs.com

2
http://simile-widgets.org/exhibit/
