# Supplementary material for: GGDonto ontology as a knowledge-base for genetic diseases and disorders of glycan metabolism and their causative genes
Source: J Biomed Semantics. 2018 Apr 18;9:14. doi: 10.1186/s13326-018-0182-0 (PMC5905134; doi:10.1186/s13326-018-0182-0)

Details of the structure of the “Pathway” classifications

The new “Pathway” classification of the congenital disorders of glycosylation (CDG) (from user interface)


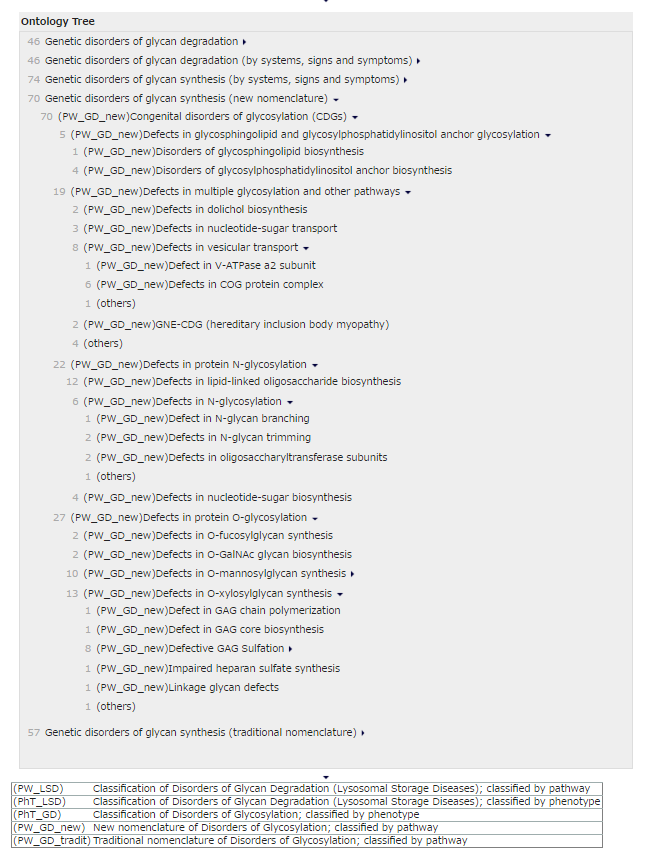


The “Pathway” classification of the lysosomal storage diseases (LSD) (from user interface)


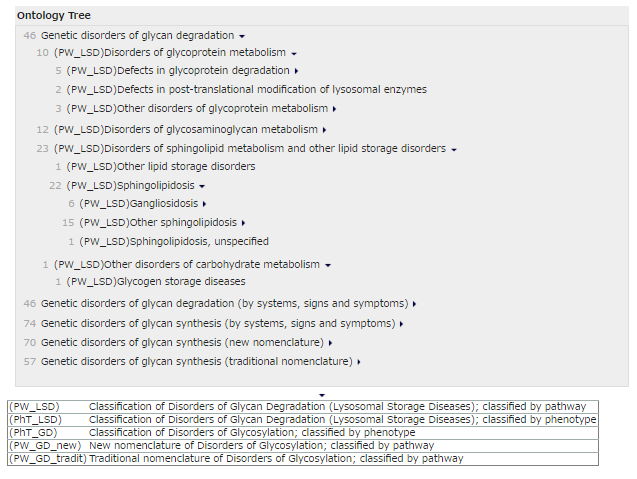

Supplement: Supplementary file 1 — Details of the structure of the “Pathway” classifications. (DOCX 161 kb) [file 13326_2018_182_MOESM1_ESM.docx]
